# Offshore medical evacuations due to non-occupational illnesses

**DOI:** 10.47626/1679-4435-2022-1033

**Published:** 2023-11-24

**Authors:** André Gustavo Matias Benevides

**Affiliations:** 1 Pós-Graduação em Medicina do Trabalho, Faculdade de Medicina, Universidade Estácio de Sá, Rio de Janeiro, RJ, Brazil; 2 Associação Nacional de Medicina do Trabalho, São Paulo, SP, Brazil

**Keywords:** occupational medicine, occupational health, absenteeism, oil and gas industry, oil and gas fields, medicina do trabalho, saúde do trabalhador, absenteísmo, indústria de petróleo e gás, campos de petróleo e gás

## Abstract

**Introduction:**

Offshore work is a continuous challenge for occupational safety and medicine, as well as for qualification, training, and worksite logistics. In such conditions, any health issue requiring disembarkation incurs a serious burden.

**Objectives:**

To evaluate the number and causes of non-occupational medical evacuations among Brazil’s offshore units between 2016 and 2019.

**Methods:**

The database of consultations performed by the medical services company International Health Care for offshore units on the Brazilian coast of client companies from 2016 to 2019 was reviewed.

**Results:**

Of the 1140 identified medical evacuations, 937 were non-occupational.

**Conclusions:**

Due to the increase in safety culture, non-occupational illness has become the most common reason for medical evacuations. Without understanding and prevention of such causes, expenses will increase for companies with offshore operations.

## Abstract (pt)

**Introdução:**

O trabalho *offshore* é um constante desafio em termos de segurança e medicina do trabalho. Outro complicador é a necessidade de maior qualificação e treinamentos específicos, fora toda a questão logística para os trabalhadores e para o local de trabalho. Com esse cenário, qualquer agravo de saúde que exija desembarque gera um enorme transtorno.

**Objetivos:**

Avaliar o volume e as causas de desembarques provocados por doenças não ocupacionais em unidades *offshore* no Brasil no período de 2016 a 2019.

**Métodos:**

Foi consultada a base de dados dos atendimentos realizados pela empresa de serviços médicos International Health Care para as unidades *offshore* na costa do Brasil de empresas clientes, no período de 2016 a 2019.

**Resultados:**

Foram identificadas 1.140 ocorrências de desembarques por motivos médicos, sendo 937 classificadas como acometimentos de saúde não ocupacionais.

**Conclusões:**

Devido à grande cultura de segurança do trabalho, o adoecimento se tornou a causa mais comum de desembarques. O não entendimento e prevenção dessas causas também causam custos às empresas.

## INTRODUCTION

Offshore oil and gas exploration and its production chain at sea occurs in units that operate 24 hours a day, 365 days a year.^1,2^ This chain includes seismic analysis, drilling, production, storage, and support vessels.^2-4^

The Campos Basin near Rio de Janeiro, Brazil, is one of the most important offshore oil production regions in the world. The estimated offshore population of workers there in 2007 was 40,000 (4,000 from Petrobras) on more than 40 oil platforms.^5^

Facility operators (or specialized companies they contract) must provide free health care services on board for any health problem, whether occupational or not, to both their own employees and outsourced workers. Units with more than 31 crew members must include at least one duly registered health professional, whether a nurse, a technician or, more rarely, a physician, who are supported onshore by a specialist physician via telemedicine, known as TopSide Support.^4,6-12^

Taking the offshore physician’s and/or TopSide Support’s opinion into consideration, best practice guidelines recommend limiting treatment in the platform’s infirmary to pathologies that will not affect work capacity for more than 24 hours.^6-12^ Workers with infectious diseases must not remain on board.^4^

Uninterrupted operation and difficult access to the facilities are a logistical challenge, regardless of whether employees are transported by air or sea (up to 35 nautical miles), which requires careful planning.^2,4,13^ For this reason offshore workers require additional qualifications, ie, in addition to having the highest skill level for their job, they must also undergo specific training, such as escaping from submerged aircraft and a basic platform safety course, in addition to other courses, depending on the company.^4^

Because the rotation is at least 14 days on/14 days off, most offshore companies prioritize skills rather than how far employees live from the departure point, which allows many to live in other municipalities or even states. Extended rotation staff (at least 21 days on/21 days off) frequently live outside the country.^8,14^ The vast majority of companies cover the travel expenses of their employees to the departure point, either by air or road, in addition to hotel stays for those must arrive the day before departure.^13-15^

Therefore, any unscheduled disembarkation is an enormous inconvenience for the company. In addition to arranging for the evacuation of the affected worker, a substitute must be located. If this substitute is on a regular rotation^15,16^ and the 1:1 ratio (eg, 14 on/14 off) is violated, any overtime receives double wages, according to article 8 of Law 5.811/72 and collective agreements with the Brazilian Offshore Workers Union (SINDITOB).

The objectives of this study were, first, to show that despite the common sense notion that the leading cause of medical leaves among offshore workers is occupational disease, it is actually non-occupational health conditions and, second, to investigate the impact of such disembarkations.

## METHODS

A retrospective cohort study was performed using the MedStatus database, which supports medical care, telemedicine, and occupational health. It was developed by a company called International Health Care to serve companies with offshore units around the world.

Reports from medical consultations that resulted in disembarkation between January 1, 2016 and December 31, 2019 in Brazil were analyzed. The medical records themselves were not analyzed, only the numbers.

In the MedStatus database, conditions are classified as occupational or not based on International Association of Drilling Contractors and International Association of Oil & Gas Producers protocols. Any injuries/accidents that occur during working hours are classified as occupational. Chronic conditions with acute worsening during a shift can also be classified as occupational.^17,18^ This distinction is always made through International Health Care’s TopSide Support and is recorded in MedStatus.^3,7^ This study did not determine whether the on board health professional was a doctor or a nurse.

Some International Health Care units operate on a 2-month contract, while others continue for years. Thus, the total population cannot be specified, only the number of visits and units served at the end of each year. The number of people on board at each unit varies according to crew capacity, as well as the stage of the operation, ranging from 15 in smaller units to more than 500.^8,19,20^

In the MedStatus report, all cases have a pathology classification (Chart 1, right column). The ICD-10 field was first incorporated into the application in 2017 (6 characters in length: eg, X00.000). Nevertheless, not all subsequent cases received an ICD-10 code. To facilitate compilation and comparison with other articles, in reports without an ICD-10 code (ie, all from 2016 and some between 2017 and 2019), the services were converted to ICD-10 codes, even if generically (Chart 1). To further simplify comparison, the ICD codes were summarized in a letter and two digits, eg, X00. The digestive system diseases group was subdivided into K00-K14 and K15-K93. Diseases of the oral cavity, salivary glands, and jaw, remained classified according to the original MedStatus category, thus maintaining emphasis on dental emergencies.

**Chart 1:** MedStatus groups according to ICD-10 classifications

ICD-10 codes	ICD-10 groups	MedStatus Groups		
A00-B99	Certain infectious and parasitic diseases	Infectious diseases		
E00-E90	Endocrine, nutritional, and metabolic diseases	Endocrinilogical		
F00-F99	Mental and behavioral disorders	Psychiatric disorder		
G00-G99	Nervous system diseases	Neurological		
H00-H59	Diseases of the eye and adnexa	Ophthalmological		
H60-H95	Diseases of the ear and mastoid process	Ear, Nose & Throat		
I00-I99	Diseases of the circulatory system	Cardiovascular		
J00-J99	Respiratory system diseases	Respiratory		
K00-K14	Diseases of the digestive tract/oral cavity, salivary glands, and jaws	Dental		
K15-K93	Diseases of the digestive system	Gastroenterological	Proctological	
L00-L99	Diseases of the skin and subcutaneous tissue	Dermatological	Ingrown toenail	
M00-M99	Osteomuscular and connective tissue diseases	Orthopedic		
N00-N99	Diseases of the genitourinary system	Genitourinary		
O00-O99	Pregnancy, childbirth, and puerperium	Gynecology/obstetrics		
R00-R99	Symptoms, signs, and abnormal clinical and laboratory findings not elsewhere classified	Backache	Pre-existing conditions	Muscle pain
S00-T98	Injury, poisoning, and certain other external causes	Trauma		

Occurrences in the ICD group R00-R99 with a specific classification in the MedStatus classification system, such as cardiological, gastroenterological, etc., were reallocated (Chart 1). Pre-existing conditions and muscle pain without a specified ICD code were grouped into ICD R00-R99, although they were not specific to a pathology or this ICD group.

There were only 2 cases classified as obstetrics/gynecology in MedStatus without an ICD-10 code. They were grouped together with the other 2 ICD R00-R99 cases also classified as obstetrics/gynecology into group ICD O00-O99 “pregnancy, childbirth, and puerperium”, rather than N00-N99 “diseases of the genitourinary system”. Since offshore work is an unhealthy and dangerous environment, pregnant women are prohibited from embarking; thus, all 4 of these patients were evacuated.^21^

During the study period, there were no occurrences of the ICD codes not shown in Chart 1, including: C00-D48 “neoplasms”, P00-P96 “certain conditions originating in the perinatal period”, V01-Y98 “external causes of morbidity and mortality”, Z00-Z99 “factors influencing health status and contact with health services”, and U00-U99 “special purpose codes”. Therefore, only the following were used as research criteria: ICD and/or MedStatus classification, date of disembarkation, and whether or not the cause was occupational. Other criteria can be assessed in subsequent studies. The analyses were performed in Microsoft Excel 365.

## RESULTS

**Figure 1:**
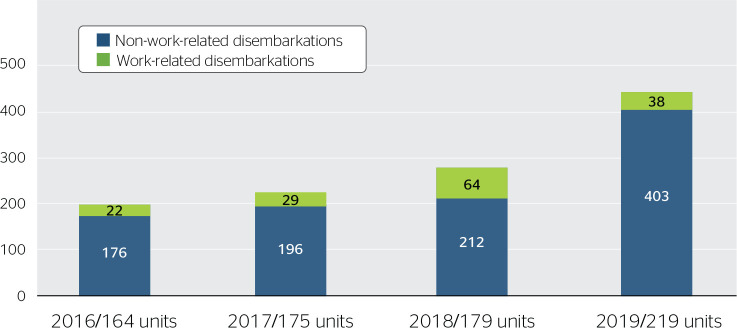
Comparison between medical evacuations for occupational and non-occupational conditions.

During the study period, 44,454 consultations were performed, of which 1140 (2.56%) led to medical evacuations. Of these, 153 (13.4%) were classified as occupational and 987 (86.6%) non-occupational. To better visualize and compare occupational and non-occupational causes of disembarkation, the consultations that did not lead to disembarkation were excluded from Figure 1. A slight increase in disembarkation occurred between 2016 and 2018, and an abrupt increase occurred in 2019. This is due to the addition of new client units as a result of expanding offshore production.

**Figure 2:**
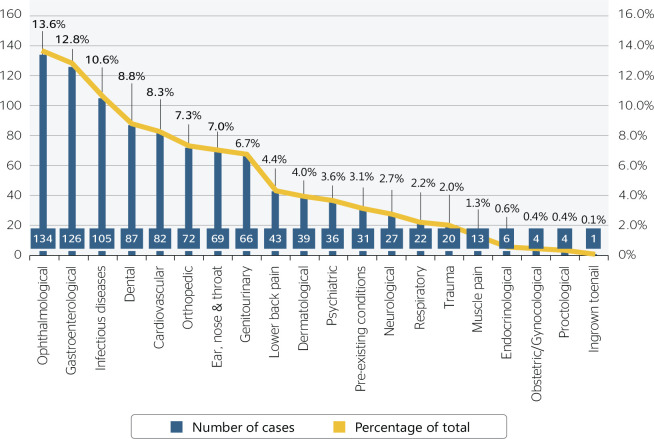
Causes of non-occupational medical evacuations between 2016 and 2019 according to MedStatus classification.

Figure 2 shows the causes of disembarkation between 2016 and 2019 according to MedStatus classification.

**Figure 3:**
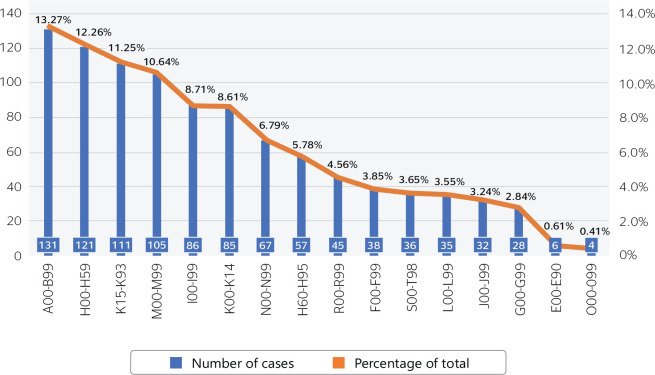
ICD-10 classification of non-occupational medical evacuations reported in MedStatus between 2016 and 2019.

Figure 3 shows the cases reclassified according to ICD-10 grouping (described in Chart 1) between 2016 and 2019.

**Figure 4:**
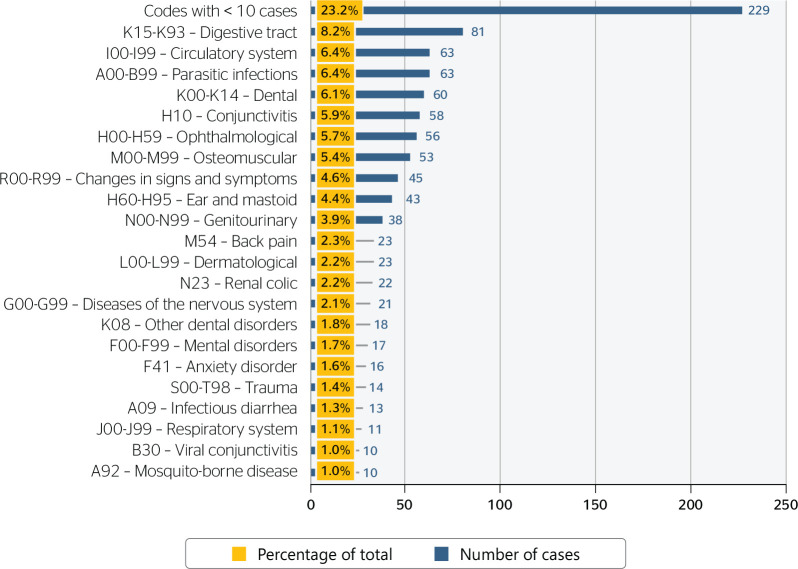
Non-occupational causes of medical evacuations reported in MedStatus between 2016 and 2019 according to ICD-10 code.

The 987 cases are distributed in 127 ICD-10 categories, of which the majority (596 [60.4%]) received a generic ICD-10 code based on the criteria in Chart 1. Before redistribution, ICD group R00-R99 “Symptoms, signs, and abnormal clinical and laboratory findings not classified elsewhere” included 107 cases. After redistribution, 45 (42.1%) remained in this group since they were classified in MedStatus as low back pain (28 cases), pre-existing conditions (14 cases), and muscle pain (3 cases), as explained above. Of these, 1 case was classified as ICD R07.4 “unspecified chest pain” and was added to the 2 muscle pain cases due to MedStatus classification. Figure 4 analyzes codes with ≥ 10 occurrences, with all others classified as “Codes with < 10 cases”. The ICD-10 code nomenclature was summarized to facilitate comprehension in this figure.

## DISCUSSION

Few health-related studies have investigated the offshore oil and gas industry, with most focusing on offshore installations in the North Sea.^9,11^ A 1996 study found that dental pathologies were the main non-occupational cause of medical evacuations in the UK.^22^ Over a period of 7 years (1988 to 1994), dental issues were responsible for an average of 12.13% of the medical evacuations.

We could only find 6 studies that considered both occupational and non-occupational causes for offshore medical evacuations, of which only 4 were similar to the present study. The 2 dissimilar studies included Smith et al.,^11^ who applied an electronic questionnaire to 352 employees from several companies, and Ponsonby et al.,^7^ who in 2009 used the 1999 United Kingdom Department of Occupational Health and Safety report to reinforce the importance of standardizing medical emergency response, improving health care training, and upgrading offshore sick bays.

Of the 4 similar studies, 2 were from the UK (published in 1988 and 1999). The study period of the first survey was between 1976 and 1984 and the second was from 1987 to 1992. Both found a downward trend in occupational disembarkations; the turning point occurred between 1988 and 1989, when non-occupational illness became the leading cause (55%) of medical evacuation. In both studies, the main non-occupational cause was dental, followed by infectious diseases (ICD groups A00 to B99, with the other cases classified as other ICD groups).^6,23^

A 2014 study on the American portion of the Gulf of Mexico found the highest ratio of non-occupational to occupational disembarkations: 304 to 93. It determined the cost of medical evacuations between 2008 and 2012 to demonstrate the financial impact of not investing in preventive health measures. The main causes of medical evacuation were cardiovascular events, which cost USD 8.8 million (not including other associated costs, such lost productivity, onshore medical coverage, and substitute employees,^9^ followed by abdominal pain and neurological causes/seizures.^9^

The latest study, from 2020, was a retrospective survey of medical evacuations in the Gulf of Thailand between 2016 and 2019 and calculated their financial impact. It also found that the majority of medical evacuations were for non-occupational causes (350 vs 66). The 416 total disembarkations were classified as non-preventable/difficult-to-prevent (60.1%) or preventable (39.9%).^12^ Infectious diseases were the main culprit, with influenza as the leading cause (84 cases [24%]). There were 24 disembarkations due to dental caries and only 10 due to cardiovascular problems. The cost of preventable diseases was calculated at USD 450,000 during the study period.^12^ The American and Thai studies stressed the importance of better admission and follow-up examinations after an illness/accident, as well as for increased treatment capacity on offshore units.^9,12^

In the present study, considering mean values of BRL 10,000 for an unscheduled seat on an offshore non-medical evacuation helicopter flight ^24^ and BRL 1500 for a last minute ticket for a national commercial flight,^26^ the cost of an unscheduled disembarkation was BRL 20,000-23,000, apart from any hotel or land transfer (airport-hotel-helicopter base) expenses. Thus, the estimated cost of the 987 medical evacuations for non-occupational illnesses was BRL 21,220,500, considering only worker logistics (the patient and the replacement) onshore and offshore. This study could was not assess a mean medical evacuation flight cost, since they are provided through annual contracts that cover different services.

## CONCLUSIONS

**Figure 5:**
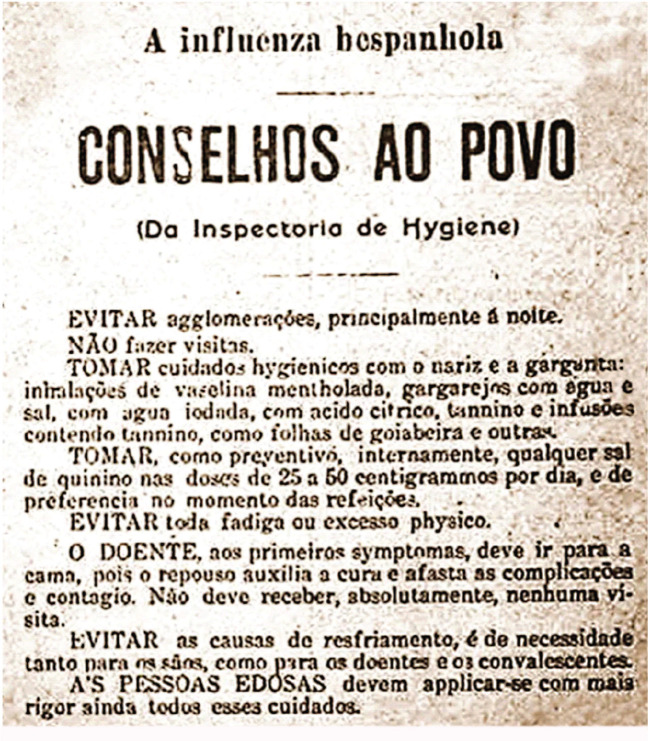
Hygiene etiquette guidelines from the São Paulo Sanitary Service during the Spanish flu epidemic, warning against social gatherings, outlining social distancing/convalescence protocols, etc^26^.

While a number of these conditions, such as appendicitis, are difficult to prevent, others, such as infectious and parasitic diseases, are preventable through health education and hygiene etiquette, which has become more commonplace today due to the COVID-19 pandemic. According to figure 5, which shows a newspaper clipping from *O Estado de São Paulo*^26^ communicating guidelines from the São Paulo Sanitary Service, hygiene etiquette was already being promoted during the Spanish flu at the end of the 1910s.

One factor that curtails on board health educational measures, such as lectures and interactive activities, is the exhausting 12-hour work routine. After their shift, most workers find it difficult to pay attention and interact in such events.^14,27^ However, a 2017 study investigated health education through apps, websites, and email messages that could be accessed at more opportune times.^28^

Another issue is the difficulty of using the ICD-10, since 43% of the occurrences between 2017 and 2019 had no defined ICD code and 9% were in the ICD Group R00-R99. This issue is not exclusive to MedStatus, constantly arising in health care.^29^

Thus, the present study, in addition to confirming the trend towards more medical evacuations due to non-occupational diseases, highlights the need for preventive measures for each target population. To accomplish this, further statistical surveys must be conducted on a local level to determine which actions should be taken.
